# A one health approach for monitoring antimicrobial resistance: developing a national freshwater pilot effort

**DOI:** 10.3389/frwa.2024.1359109

**Published:** 2024-05-17

**Authors:** Alison M. Franklin, Daniel L. Weller, Lisa M. Durso, Mark Bagley, Benjamin C. Davis, Jonathan G. Frye, Christopher J. Grim, Abasiofiok M. Ibekwe, Michael A. Jahne, Scott P. Keely, Autumn L. Kraft, Betty R. McConn, Richard M. Mitchell, Andrea R. Ottesen, Manan Sharma, Errol A. Strain, Daniel A. Tadesse, Heather Tate, Jim E. Wells, Clinton F. Williams, Kim L. Cook, Claudine Kabera, Patrick F. McDermott, Jay L. Garland

**Affiliations:** 1 United States (U.S.) Environmental Protection Agency, Office of Research and Development, Cincinnati, OH, United States,; 2 U.S. Centers for Disease Control and Prevention, Atlanta, GA, United States,; 3 U.S. Department of Agriculture, Agricultural Research Service (USDA, ARS), Agroecosystem Management Research, Lincoln, NE, United States,; 4 USDA ARS, U.S. National Poultry Research Center, Poultry Microbiological Safety and Processing Research Unit, Athens, GA, United States,; 5 Center for Food Safety and Applied Nutrition, U.S. Food and Drug Administration, College Park, MD, United States,; 6 USDA, ARS, Agricultural Water Efficiency and Salinity Research Unit, Riverside, CA, United States,; 7 Oak Ridge Institute for Science and Education, USDA, ARS, Beltsville, MD, United States,; 8 Environmental Protection Agency, Office of Water, Washington, DC, United States,; 9 Center for Veterinary Medicine, National Antimicrobial Resistance Monitoring System (NARMS), U.S. Food and Drug Administration, Laurel, MD, United States,; 10 USDA, ARS Environmental Microbial and Food Safety Laboratory, Beltsville, MD, United States,; 11 USDA, ARS, U.S. Meat Animal Research Center, Meat Safety and Quality, Clay Center, NE, United States,; 12 USDA, ARS, US Arid-Land Agricultural Research Center, Maricopa, AZ, United States,; 13 USDA, ARS Nutrition, Food Safety and Quality National Program Staff, Beltsville, MD, United States

**Keywords:** antimicrobial resistance, surface waters, monitoring, one health, freshwater, environment, human health

## Abstract

Antimicrobial resistance (AMR) is a world-wide public health threat that is projected to lead to 10 million annual deaths globally by 2050. The AMR public health issue has led to the development of action plans to combat AMR, including improved antimicrobial stewardship, development of new antimicrobials, and advanced monitoring. The National Antimicrobial Resistance Monitoring System (NARMS) led by the United States (U.S) Food and Drug Administration along with the U.S. Centers for Disease Control and U.S. Department of Agriculture has monitored antimicrobial resistant bacteria in retail meats, humans, and food animals since the mid 1990’s. NARMS is currently exploring an integrated One Health monitoring model recognizing that human, animal, plant, and environmental systems are linked to public health. Since 2020, the U.S. Environmental Protection Agency has led an interagency NARMS environmental working group (EWG) to implement a surface water AMR monitoring program (SWAM) at watershed and national scales. The NARMS EWG divided the development of the environmental monitoring effort into five areas: (i) defining objectives and questions, (ii) designing study/sampling design, (iii) selecting AMR indicators, (iv) establishing analytical methods, and (v) developing data management/analytics/metadata plans. For each of these areas, the consensus among the scientific community and literature was reviewed and carefully considered prior to the development of this environmental monitoring program. The data produced from the SWAM effort will help develop robust surface water monitoring programs with the goal of assessing public health risks associated with AMR pathogens in surface water (e.g., recreational water exposures), provide a comprehensive picture of how resistant strains are related spatially and temporally within a watershed, and help assess how anthropogenic drivers and intervention strategies impact the transmission of AMR within human, animal, and environmental systems.

## 1 Introduction

Antimicrobial drugs have been widely used in human and veterinary medicine and agroecosystems for more than 80 years, with tremendous benefits to human, animal, and plant health. However, the use of antimicrobials also represents an evolutionary selective pressure on microbes (Aminov, 2010), and prolonged use and/or overuse in a particular environment can lead to alterations in the presence of antimicrobial resistant strains within a microbial community (e.g., increases or decreases of resistance naturally found in the population, evolution of new resistance, etc.). Once alterations in resistance have occurred, the genes conferring resistance can spread to other species through horizontal transfer of mobile genetic elements (MGEs) (Baharoglu et al., 2013; Marti et al., 2014), or via clonal spread of bacteria that carry the resistance element (Baker et al., 2017). In addition to antimicrobials, other stressors can mobilize MGEs, such as heavy metals, oxidative stress, and ultraviolet light. This can lead to co-selection of both antimicrobial resistance genes (ARGs) and other stress-response genes (e.g., heavy metal resistance genes) (Poole, 2012; Pal et al., 2015, 2017). Over time, these selective pressures have led to the development of highly resistant human pathogens such as Methicillin-resistant *Staphylococcus aureus* and extreme drug-resistant tuberculosis, that are difficult to treat (O’Neill, 2016).

As existing antimicrobials become less effective due to the emergence of antimicrobial resistant bacteria (ARB), the risks associated with bacterial infections (e.g., following surgery or chemotherapy) increase. The global burden of antimicrobial resistance (AMR) was estimated at 4.95 million deaths in 2019, with 1.27 million of those deaths directly caused by resistant infections (Murray et al., 2022). It’s predicted that the deaths attributable to AMR infections will increase to 10 million globally by 2050 (O’Neill, 2016). The World Health Organization (WHO) has also identified AMR as one of the leading global health threats (World Health Organization, 2000).

To effectively mitigate the threat of AMR, scientific researchers, health professionals, and government agencies must collaborate in new ways. The concept of One Health has been adopted to address the challenge of AMR given that the same antimicrobials are used in human and animal medicine as well as agriculture, and humans and animals can harbor the same pathogens. The One Health paradigm recognizes that human and animal health are linked to environmental health, and that there is a need to better understand the role of the environment in disease ecology and transmission. The United States (U.S.) Centers for Disease Control and Prevention (CDC) defines One Health as *a collaborative, multisectoral, and transdisciplinary approach — working at the local, regional, national, and global levels — with the goal of achieving optimal health outcomes recognizing the interconnection between people, animals, plants, and their shared environment* (U.S. Centers for Disease Control and Prevention (CDC), 2023). A One Health approach for AMR recognizes the need for a holistic system to combat antimicrobial resistance that encompasses human, animal, and plant health and the role of the environment in mediating the spread of AMR. This One Health approach also involves the development of collaborative systems for effectively monitoring the emergence and movement of resistance genes and resistant bacteria within and between biological compartments.

An early AMR monitoring effort was established in the U.S. in the mid 1990’s when enrofloxacin was approved for use in poultry. This use of enrofloxacin raised concerns about the transmission of fluoroquinolone resistant bacteria through the food system (Tollefson et al., 1998). As a result, in 1996, the U.S. Food and Drug Administration (FDA), the CDC, and the U.S. Department of Agriculture (USDA) collaborated to establish the National Antimicrobial Resistance Monitoring System (NARMS). NARMS was designed to detect and track AMR in foodborne and other enteric bacteria, like *Salmonella, Campylobacter, E. coli, Enterococcus*, etc., isolated from human and animal clinical cases, food, and food animal processing environments (U.S. Food and Drug Administration (FDA), 1994a,b, 2000). As the foundational and main system currently used to monitor AMR in the U.S. food system, NARMS provides key data on which research and policy decisions are based.

In 2000, WHO released a report drawing attention to AMR as a global health threat (World Health Organization, 2000). In 2015, WHO adopted the Global Action Plan on AMR, which urged the international community to establish national monitoring systems to assess AMR in bacteria isolated from both humans and animals and underscored the need to adopt a One Health approach (World Health Organization, 2015). Concomitant with the adoption of a One Health approach to mitigating AMR was a growing realization that understanding the ecology, evolution, and epidemiology of AMR and ARB infections requires integrating data from multiple sources and disciplines (National Academies of Sciences (NAS), Engineering, and Medicine, 2017; Topp, 2017; McEwen and Collignon, 2018).

Although the NARMS program has developed data on AMR in human and food-animal systems, information on AMR in the environment (such as surface waterways, soil, or wildlife) (Marti et al., 2014; Barrett and Bouley, 2015) is more limited. Following the 2017 FDA’s Science Board recommendation that NARMS pursue an integrated, One Health approach, a need for baseline data on AMR in the environment was identified. As a result, the establishment of a geographically representative monitoring system for AMR in the environment was added as a goal to the NARMS Strategic Plan: 2021–2025 with the intent of building off of previous work performed by the U.S. Environmental Protection Agency (EPA) analyzing select ARGs in surface waters nationwide (Keely et al., 2022). An environmental working group (EWG) coordinated by the EPA, FDA, CDC, and USDA Agricultural Research Service (ARS) was formed in 2020 to establish an initial environmentally based monitoring system within NARMS.

## 2 Background on environmental AMR monitoring

### 2.1 Current status of environmental AMR monitoring

The importance of monitoring AMR in the environment has stimulated multiple discussions and review articles on the best sampling and laboratory methods (e.g., Berendonk et al., 2015; Franklin et al., 2016; Matheu et al., 2017; Larsson et al., 2018; Ben et al., 2019; Huijbers et al., 2019; Diallo et al., 2020; Larsson and Flach, 2021; Pruden et al., 2021; Kaiser et al., 2022; Liguori et al., 2022). These reports outlined key components necessary for environmental AMR monitoring, current knowledge gaps, and limitations of the methods currently used to monitor environmental AMR. A common theme across these reviews is that the ideal environmental AMR monitoring system should be part of a larger effort that also monitors AMR in human and animal populations (i.e., a One Health approach).

A systematic literature review of publications that described AMR monitoring programs across 35 countries found that 65 of the 71 programs monitored AMR in bacteria isolated from humans, while 18 monitored AMR in bacterial isolates from animals and none monitored AMR in bacterial isolates from the environment (Diallo et al., 2020). Similarly, Kaiser et al. (2022) reviewed 25 National Action Plans (NAP) for AMR monitoring and used a One Health lens when analyzing each plan’s priorities. In general, the NAPs did not incorporate environmental monitoring, or only incorporated environmental components when they directly related to human exposures. While most environmental AMR research has been reactive to known environmental contamination, limited proactive strategies for managing ARB in the environment have been identified (Wellcome Trust, 2020; Kaiser et al., 2022). These studies highlight the fact that most existing AMR monitoring systems do not include environmental monitoring even though there is widespread consensus within the scientific community that a One Health approach is the optimal way to monitor AMR. As such, the establishment of environmental AMR monitoring systems, like the NARMS environmental monitoring effort presented herein, represent a key gap and critical need.

Even among existing human and animal AMR monitoring efforts, harmonization of methods and international collaboration is lacking (Diallo et al., 2020; Haenni et al., 2022). The sampling, laboratory methods, and data management approaches employed by different monitoring systems are not harmonized, and as a result the data produced may be difficult to compare. Sample sizes and sampling designs differ between monitoring systems, with some efforts performing selective sampling while others are sub-sampling entire populations (Chau et al., 2022). Laboratory methods similarly vary, including the type of bacterial indicator, antibiotic compounds used for susceptibility testing, and the monitored phenotypes and genotypes. In some instances, different antibiotics are used to define the same phenotype or genotype since the same genetic determinants can provide resistance to multiple antibiotics (Diallo et al., 2020). Even when the same bacterial indicators, antibiotic compounds, and/or phenotypes are monitored, different methods may be used for bacterial isolation and susceptibility testing. Lastly, data and metadata collection and management vary between monitoring efforts. Inconsistencies in the type and method of metadata collected may severely limit the international comparability of data from different monitoring systems, as well as the utility of these data for guiding public health decisions. Overall, monitoring efforts should use sampling and laboratory methods that align and provide comparable data, and, then, the data produced should be collected and managed in a manner that ensures coordination across space, time, and biological compartments, ideally within a standardized framework.

The NARMS EWG divided the development of the environmental monitoring effort into five areas: (i) key objectives and questions, (ii) optimal study/sampling design, (iii) selection of AMR indicators, (iv) selection of methods, and (v) development of data management/analytics/metadata plan. For each of these areas, the consensus among the scientific community and literature was reviewed and carefully considered prior to the development of this environmental monitoring program. The remainder of this paper outlines key aspects for each of these five areas, and then defines the specific implementation of the pilot environmental monitoring effort accordingly.

### 2.2 Current recommendations for AMR monitoring in the environment

#### 2.2.1 Determine key objectives and questions

When designing a new monitoring system, the objectives and key questions being posed should drive the overall study design. For the development of an environmental AMR monitoring program, the role that the environment plays in AMR-related processes is key for defining these objectives and key questions. The environment can serve two primary roles in AMR-related processes; first, to disseminate already resistant bacteria and genes within and between humans and animals, and second, as a source and facilitator for the evolution of AMR (Bengtsson-Palme et al., 2023). Anthropogenic activities can actively shape and alter environmental resistomes, especially in polluted water bodies. Although evidence is sparse thus far, recent research has found that the environment can be directly attributable to human colonization by resistant bacteria (Leonard et al., 2018, 2022) as well as resistant infections in clinical settings (Stanton et al., 2022). However, the relative contributions of different environmental AMR sources (e.g., untreated human versus animal waste) to infections in humans with immediate epidemiological linkages is still unclear. Similarly, the concentrations and/or mixtures of environmental factors and pollutants (physicochemical, pharmaceutical, heavy metals) that would significantly elevate selective pressures for the maintenance of resistance in the environment is unknown. Furthermore, the levels or concentrations of AMR in surface waters that would pose an increased exposure risk to humans is still an open question (Niegowska et al., 2021).

Aligning the objectives and key questions of an environmental AMR monitoring program with current AMR monitoring programs looking at human, animal, and food systems, like NARMS, is essential for creating a One Health assessment of AMR. For example, the data gathered by NARMS from foodborne and enteric bacteria within humans, animals, and food systems can be used for source attribution of enteric illnesses, investigation of underlying genetic mechanisms of resistance, an early warning system for emerging threats, and ultimately guiding public health efforts in the prevention of resistant infections through the judicious use of antimicrobials (Karp et al., 2017). While the immediate linkages to intervention measures is more difficult within environmental systems, the purpose of environmental AMR monitoring fall into several similar categorical objectives: (i) track the rates of resistance over time in key environments and organisms, (ii) determine the sources and drivers of environmental AMR, (iii) monitor for the evolution of new resistance mechanisms, and (iv) determine the exposure risks posed to humans for colonization/infection in impacted environments. These objectives can be achieved in several different monitoring schemes, and each are dependent upon analytical methodology, budgetary constraints, and scope of the proposed monitoring system.

#### 2.2.2 Sampling design

Once the objectives of the monitoring system are determined, the sampling design can be devised, piloted, and scaled appropriately. For example, if the objective is to characterize the baseline presence of AMR within a certain environment (e.g., river systems, soil) at a large scale (e.g., nation-wide) then a probabilistic sampling design would be adequate since it randomly selects sampling locations to represent the overall population of interest without creating sample biases. On the other hand, if the objective is to identify drivers and areas with significant AMR hazards (e.g., AMR hot spots), then environmental sampling locations need to be selected using prior knowledge of possible AMR point sources that could facilitate transmission of ARB between humans, plants, and animals. The scale of the study may also affect sampling considerations since it is much easier to implement a targeted sampling plan at a regional scale than a general sampling scheme at a national level as it requires in-depth knowledge about local processes and land uses.

A common limitation of sampling schemes designed to monitor environmental AMR is the absence of extensive, high-frequency, time-series datasets, especially in surface waters. These types of sampling designs not only establish baseline data for the examination of environmental AMR but also facilitate the identification of acute drivers of AMR through seasonality, random events, and/or other unknown factors. These timeseries datasets are best applied at critical control points where known anthropogenic inputs introduce genes and bacteria into the system. These control points include domestic and industrial wastewater treatment plants effluents (Pazda et al., 2019), hospital effluents (Paulus et al., 2019), high-density and/or older/failing septic system areas (Junaid et al., 2022), combined sewer overflow and urban runoff outfalls (Almakki et al., 2019), and high-density agricultural areas and CAFO runoff sites (Lopatto et al., 2019). In conjunction, regular monitoring of known exposure sites/routes such as impacted recreational water bodies and any groundwaters (e.g., private well users) that may be affected by these pollution sources, will allow the characterization of infection/colonization risks.

Additionally, the development of a new monitoring program (and/or research study) could be designed along a good, better, best spectrum, as outlined by (Harris et al., 2013). During the initial planning phase of a large-scale project, it may not always be clear what limitations may exist (e.g., funding availability, supply chain issues, laboratory capacity, ethical considerations, etc.). Therefore, identifying the generally accepted good, better, and best practices for each element of the study and sampling design will aid in making final decisions once funding sources, laboratory capacity, availability of supplies, etc. are known.

#### 2.2.3 Selection of AMR indicators

For a One Health environmental monitoring system focused on ARB, selection of AMR indicators (e.g., bacteria, genes, antibiotics, etc.) should be guided by existing recommendations from the WHO and other public health organizations as well as local behaviors (e.g., which antimicrobials are commonly used to treat humans and livestock in the region where the study is being conducted). Indicators should also be selected to facilitate investigations into the transmission of AMR within and between human and animal populations, and the environment to inform possible mitigation strategies. Therefore, overall selection of indicators should be based on relevance for humans, animals, and environment in question, the feasibility of collecting and analyzing samples for that indicator, and sensitivity to change within the prescribed time frame of monitoring. To improve and broaden information about AMR in the environment, baseline lists of ARB and ARG indicators that should be used across AMR monitoring efforts have been suggested (Haenni et al., 2022). For example, a commonly suggested ARB indicator is *Escherichia coli* (*E. coli*) resistant to 3rd generation cephalosporins (3GC). The use of 3GC-resistant *E. coli* as an indicator for environmental AMR monitoring is supported by the WHO extended-spectrum beta-lactamases (ESBL) *E. coli* “Tricycle protocol” (Anjum et al., 2021; World Health Organization, 2021), and logical given the widespread use of 3GC in human and veterinary medicine (Temkin et al., 2018; U.S. Food and Drug Administration (FDA), 2018; European Medicines Agency (EMA), 2019). In addition, *Enterococcus* spp. (vancomycin resistant) have been proposed as a convenient gram-positive counterpart to *E. coli* given their extensive use as a water quality indicator for decades (U.S. Environmental Protection Agency (EPA), 2012; Holcomb and Stewart, 2020; Liguori et al., 2022). Identifying absolute and relative values (i.e., CFU/mL and percentage of resistant colonies) of these ARB provide useful information for assessing human and animal exposure rates to environmental sources of AMR and identifying hotspot*s* in the environment.

Selection of ARGs should include clinically relevant and anthropogenically sensitive genes that commonly occur in freshwater sources and take into consideration factors such as abundance of the gene, propensity for lateral transfer, and ability of ARGs to be expressed in pathogens (Berendonk et al., 2015; Ashbolt et al., 2018; Nnadozie and Odume, 2019; U.S. Centers for Disease Control and Prevention (CDC), 2019; Keenum et al., 2022; Zhang et al., 2022). For example, *bla*_CTX-M_ and *vanA* have been recommended as clinically relevant ARGs, since the types of resistance that these ARGs confer to pathogens are noted as “serious” concerns on the CDC threat list (U.S. Centers for Disease Control and Prevention (CDC), 2019). *Bla*_CTX-M_, which encodes for ESBL, is responsible for therapeutic problems, and *vanA* encodes resistance to vancomycin, a last resort antibiotic for treatment of enterococcal infections. Additionally, *sulI* and *tetA* are ARGs that tend to be associated with anthropogenic sources with *sulI*, typically carried by class 1 integrons, conferring resistance to sulfonamides and *tetA* encoding resistance to tetracyclines, a widely used antibiotic by humans and livestock (Yoshizawa et al., 2020). Besides ARGs, *intI1*, an integron-integrase, is commonly used as a marker of anthropogenic pressure and/or pollution with higher abundance associated with waste streams and lower in more pristine environments (Gillings et al., 2015; Lucassen et al., 2019; Keely et al., 2022). Its environmental presence, particularly in surface water, is often correlated with the presence of ARGs because integrons are genetic mechanisms that allow bacteria to adapt and evolve rapidly through the stockpiling and expression of new genes (e.g., through site-specific recombination) (Gillings et al., 2015). Coupling the analysis of *intI1* with ARGs can provide insights into ARG mobility in environmental systems.

A final set of critical indicators for AMR monitoring efforts in the environment are antimicrobial compounds like antibiotics and other stressors, such as metals and pesticides (Huijbers et al., 2019). Their utility as bioactive compounds are known to create selective pressure for evolution, selection, and maintenance of AMR in bacteria, even at environmentally relevant concentrations (Sandegren, 2014). Antimicrobials in the environment also pose a potential risk to terrestrial and aquatic ecosystem health if they are present at concentrations that alter microbial community function and structure (e.g., nitrification, denitrification, anaerobic ammonium oxidation inhibition). Analysis of antimicrobials in the environment, particularly water, can provide insights into the use of antibiotics in human and animal populations and thereby allow for monitoring of its potential association with observed AMR indicators (Pärnänen et al., 2019). Simultaneous monitoring of antibiotics and AMR is recommended for ensuring continuity and comparability across efforts and maximizing data utility to end-users. Human health, animal health, and environmental health organizations each have developed lists of priority drug indicators to monitor and include fluoroquinolones, sulfonamides, tetracyclines, trimethoprim, and aminoglycosides (World Health Organization, 2018; Gomez Cortes et al., 2020; Haenni et al., 2022). However, most environmental monitoring efforts are not analyzing for antibiotics or other selective agents likely due to the number of antimicrobials that would need to be monitored, lack of technical harmonization and optimization of detection methods, difficulty detecting low levels of antimicrobials in environmental matrices, and/or costs associated with these analyses (Niegowska et al., 2021).

#### 2.2.4 Selection of methods

Once appropriate AMR indicators are selected, analytical methods need to be identified. A combination of culture-based and culture-independent methods provide a comprehensive analysis of AMR in the environment (Franklin et al., 2016, 2021; Niegowska et al., 2021; Pruden et al., 2021). Culturing bacteria and performing standardized *in vitro* antimicrobial susceptibility testing has been a cornerstone of AMR monitoring since the beginning of the antibiotic era in medicine. This methodology feeds directly into the goals of a One Health approach for AMR by detecting and characterizing ARB that can potentially cause human and animal disease. However, when looking at environmental microbiomes for a comprehensive picture of resistance, this approach is inadequate. Only a small subset of environmental bacteria can be cultured in a laboratory setting, and determination of phenotypic resistance for environmental bacteria is limited by what susceptibility testing can be performed (e.g., availability of validated methods, laboratory capacity, etc.). Furthermore, the diversity of the gene pool for environmental bacteria is much larger compared to bacteria associated with humans or domestic animals, creating a wider array of genetic traits, including novel ARGs (Panthee et al., 2022). The inclusion of molecular analysis of AMR (targeted gene analysis, metagenomics, and whole genome sequencing) can provide information about the entire bacterial population and the environmental resistome of each sample that would otherwise be missed with culture-based analysis alone. A comprehensive molecular method approach can identify and/or quantify known ARGs and MGEs through targeted gene analysis as well as discover emerging or novel forms of resistance with non-targeted techniques like metagenomics and/or whole genome sequencing (Franklin et al., 2021). If monitoring *E. coli* and *Enterococcus* bacteria and fecal indicator genes both culture and molecular analysis can also be used to measure fecal contamination, which provides information about the potential for transmission and evolution of AMR (Liguori et al., 2022).

The use of standard methods within and across multiple monitoring efforts is needed to ensure consistency across laboratories (Berendonk et al., 2015; Franklin et al., 2016; Liguori et al., 2022) so that results will be comparable across studies and monitoring efforts. While standard methods are readily available for analysis of AMR in human and animal clinical samples, these methods are not always compatible with the complex matrices of environmental samples. Several recommendations from governmental and non-governmental groups on the best methods to use in detecting certain indicators have been proposed. For example, the WHO is currently recommending the Tricycle protocol for analyzing ESBL *E. coli* in surface waters, wastewaters, human, and animal samples (World Health Organization, 2021). While a recent U.S. effort funded by the Water Research Foundation has recommended a modified mTEC method (modification of EPA standard method 1603, U.S. Environmental Protection Agency (EPA), 2014; Liguori et al., 2022) and a modified mEI method (modification of EPA standard method 1600, U.S. Environmental Protection Agency (EPA), 2009; Davis et al., 2022) for the analysis of resistant *E. coli* and *Enterococci*, respectively, in surface waters, wastewaters, and reused waters.

#### 2.2.5 Development of data management/analytics/metadata plan

Obtaining pertinent key metadata is crucial for interpreting AMR data as well as for use in subsequent models to determine key drivers and risks of AMR in environmental, human, and animal sectors. Metadata is broadly defined as the contextual information about data, but for most biological studies, this refers to basic descriptive information like geographic location, sample type, and sampling date. The type of metadata collected and the method of collection need to be carefully considered when establishing any monitoring effort. The specific metadata that should be collected is dependent on the system being analyzed or monitored. Key metadata categories have been deemed important for environmental efforts, such as climate information, water quality, geographical information, watershed information, and sampling methodologies (Sano et al., 2020). Currently the curation of metadata and knowledge from monitoring systems and published literature is a challenge in the assessment of AMR and the ability to compare across systems (McArthur and Wright, 2015). Therefore, having clear, standardized metadata management, including metadata collection, cleaning, storage, and nomenclature is important for sharing data across studies and time frames.

First and foremost, metadata collection ensures the preservation of contextual information. Careful management and stewardship also ensure accuracy, consistency, privacy/confidentiality concerns, and access to metadata. Indeed, a sampling site’s GPS coordinates are considered critical metadata; if the coordinate reference system for the coordinates is not recorded and linked to the GPS data then those coordinates cannot be reliably used for linking the water quality data with other spatial metadata or when using the GPS coordinates for follow-on meta-analyses. Similarly, slight variations in the way a given parameter is measured by different studies can affect comparability; for instance, data generated by studies that use total suspended solids to track sediment levels are not comparable to data generated by studies that measure turbidity. These considerations are particularly important as there is an increasing interest in reusing data (and associated metadata) for meta-analysis and other research outside the scope for which the data were originally collected. In the context of this evolving interest, it is paramount that metadata collection and management is standardized and harmonized in a way that facilitates re-use and is amenable to the use of machine learning, artificial intelligence, and other big data analytical approaches. This impetus was a driving factor behind the establishment of the FAIR (Findable, Accessible, Interoperable, Reusable) guiding principles (Wilkinson et al., 2016).

Just as research studies can be designed along a good, better, best spectrum (Harris et al., 2013), the same principles can be applied to metadata. A good metadata system would be comprehensive, while a better system would be standardized and contain controlled vocabularies and taxonomies. Controlled vocabularies and taxonomies can be thought of as pick lists of terms that are accepted for a certain variable (Hedden, 2010). The best system would be one that has maximum re-use potential, conveying rich contextual data in a structured, machine-readable format. Ontologies are formal and standardized terms that describe objects or data in a particular setting, similar to controlled vocabularies, and additionally their relationship to each other, in a hierarchical system. Ontologies can also and often do share vocabularies, thereby further connecting and layering contextual information across studies and disciplines. This additional layer, or layers, of information enable even more complex queries of research data.

One approach to managing data is the inclusion of data management or stewardship plans, which are becoming more common and increasingly required by funding agencies. Metadata standards serve an analogous purpose for metadata. These standards, or schema, establish a structured and organized way to manage metadata. A growing list of metadata standard packages and models are available, with some disciplines offering several choices (Yilmaz et al., 2011; Delgado et al., 2018; Harrison et al., 2018).

## 3 Development of the surface water antimicrobial resistance monitoring system (SWAM)

### 3.1 SWAM study design: objectives and sampling plan

Surface waters were selected as the preferred matrices to monitor and profile AMR since water creates a conduit for environmental transmission of AMR microbes between humans, animals, and the other environments. The overall objective of the newly designated Surface Water Antimicrobial Resistance Monitoring System (SWAM) was to profile AMR in bacteria from freshwater surface waters (i.e., a watershed) as an initial environmental component within a One Health focused NARMS program. The EWG defined four main primary uses for these data: (i) generate baseline data on AMR in U.S. surface waters, (ii) perform quantitative risk assessment for AMR associated with various water uses (e.g., recreational, drinking, agricultural), (iii) characterize drivers of AMR occurrence and selective pressures that facilitate the emergence, spread, and persistence of AMR, and (iv) identify critical control points for managing AMR hazards in surface water systems.

**FIGURE 1:**
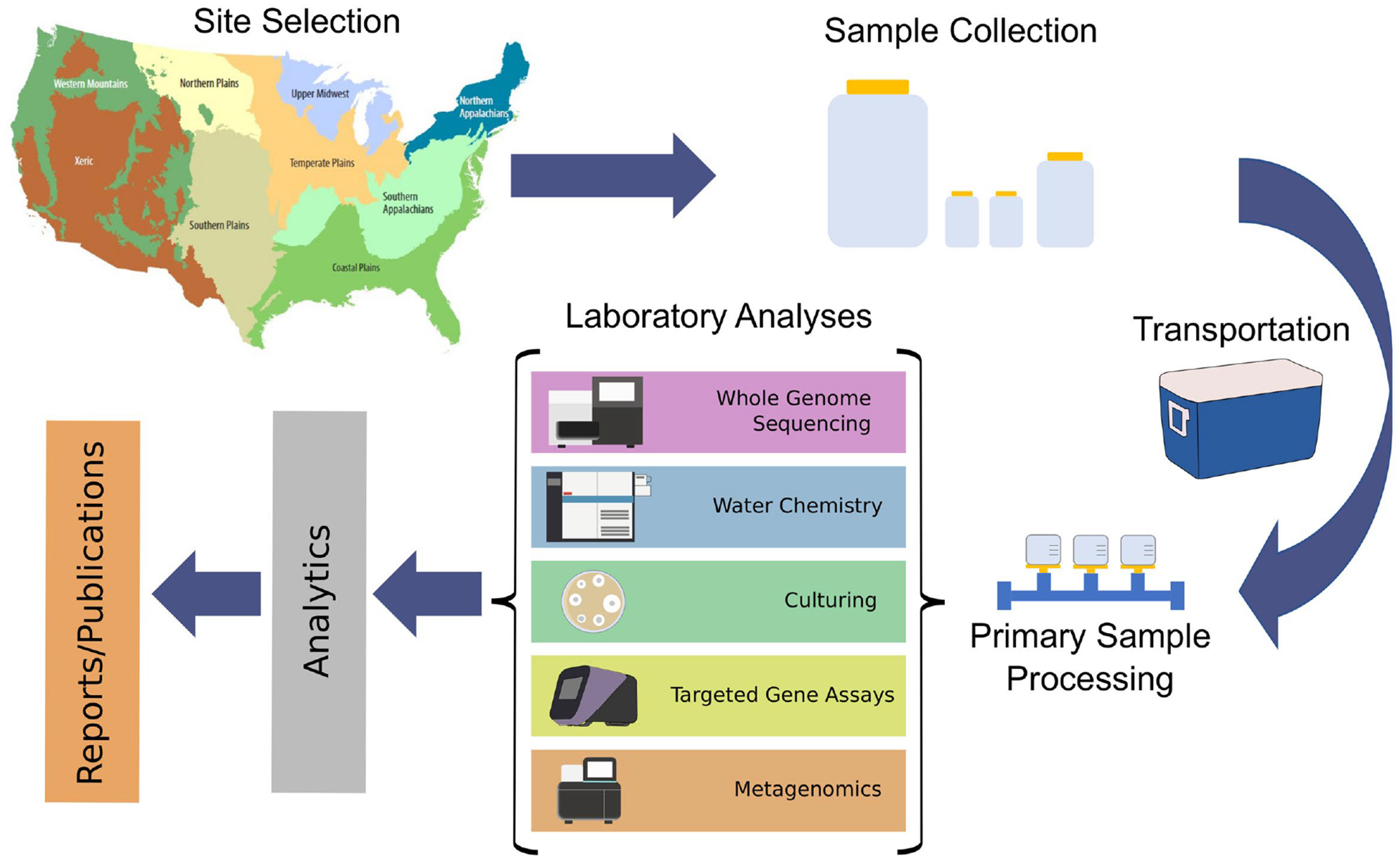
Schematic of an environmental monitoring effort for antimicrobial resistance in the environment.

To coordinate the establishment of a national surface water monitoring system, task-oriented subgroups were formed from the EWG membership to develop study designs, standardized sampling, laboratory and data management decisions and protocols, and data use plans (Figure 1; Table 1). For example, the *End Use of the Data Group* provided an interface with the NARMS program, which helped resolve issues related to integration with existing NARMS reporting structures and ensured that the data collected met user needs. Specifically, the *End Use of the Data Group* aimed to answer (i) what are the key insights and outputs desired from SWAM, (ii) how will and could the SWAM data be used to support modeling and quantitative risk assessment, (iii) how do the SWAM data link with data collected by other monitoring programs, such as NARMS and the National Rivers and Streams Assessment (NRSA), an EPA program that monitors water quality.

After an initial planning period, the EWG convened a summit so that each subgroup could share their proposals for the respective elements of the new national monitoring system. A specific focus of these proposals was the ability to provide robust data on environmental AMR that aligned with NARMS priorities and data reporting. Overall, this meeting provided an integrated assessment of the system’s scope and needs, including what data and metadata needed to be collected and how this data would be managed and used. Given the large scale of the SWAM effort, a phased approach was adopted for implementing the national monitoring system. The five phases were (i) Method Development Evaluation and In-Lab Validation; (ii) Field Validation of Methods in a Single Watershed Pilot Study; (iii) a Probabilistic National Study; (iv) Finalized National Monitoring Program; and (v) Additional Focused Studies to Address Specific Research Needs (see Table 2 for objectives of each phase).

Multiple sampling designs were evaluated to determine which could best fit the proposed goals of the surface water pilot (see Table 3 for surface water pilot goals). However, no single study could adequately capture the requirements for providing a quantitative assessment of AMR at a national scale while also providing insight into local scale dynamics, including AMR drivers and selection pressures needed to inform risk models and mitigation strategies. To circumvent these problems, a “hybrid” sampling design was selected, entailing both extensive national sampling and intensive watershed scale sampling, which would provide insight on both national trends and watershed scale dynamics. As suggested by World Health Organization (2021) and others, design of both the national-scale and watershed scale components aimed to leverage existing environmental monitoring programs for cost efficiency and to ensure that they provide contextual environmental data. Various national monitoring programs that were explored, which included U.S. Geological Survey’s National Water Quality Assessment (NAWQA; Gilliom et al., 1995), National Science Foundation’s National Ecological Observatory Network (NEON, 2011), USDA’s Conservation Effects Assessment Project (CEAP; Duriancik et al., 2008), EPA’s NRSA (U.S. Environmental Protection Agency (EPA), 2020), and US Army Corps of Engineers’ Water Quality Program for reservoirs (Medina et al., 2019). These programs were evaluated for a variety of factors related to AMR monitoring, including the sample population, the sampling density and frequency, their ability to integrate AMR sampling methods, and associated costs.

The EPA’s NRSA was chosen for the national scale study because it utilizes a spatially stratified probabilistic design with the objective of providing an unbiased population assessment of rivers and streams across the 48 contiguous states and 9 distinct ecoregions. With over 1,800 sites included in the survey, the target sampling locations include a wide range of perennial flowing waters from headwater streams to the largest rivers and catchments in the U.S., representing over 1.2 million river and stream miles. Given the natural variation in biological and chemical water quality indicators across the country, an integral part of the study design is the demarcation of strata (state, ecoregion, and river and stream size) which allows for the identification of least-disturbed reference sites that are regionally relevant (U.S. Environmental Protection Agency (EPA), 2020). These reference sites can then be used to identify drivers of environmental AMR at the national scale and across macroecological boundaries. Of note, while Alaska and Hawaii are not included in the overall study design due to differing climates, shipping limitations, and monetary restrictions, smaller scale projects may be performed in those states.

The East Fork Little Miami River (EFLMR) in southeastern Ohio was selected for the pilot watershed study because an established surface water monitoring study was already in place since 2006 to assess nutrient inputs and management (Peed et al., 2011; Schenck et al., 2015; Scown et al., 2017) and it is within proximity of EPA’s research facility in Cincinnati, OH. The watershed encompasses 1,295 km^2^ and is primarily agricultural (64%) but grades into suburban and urban areas closer to Cincinnati. Septic systems, many failing, are abundant in rural areas while wastewater treatment plants of varying capacities are situated near smaller population centers (Ohio EPA, 2021). Harsha Lake, an 8 km^2^ reservoir that includes two recreational beaches and the intake for a drinking water plant, is downstream of many of these effluents. Since any one watershed can only possess a subset of characteristics that are important for characterizing AMR, it is important to build out a series of watershed studies over time to complement the national probabilistic survey. For example, it will be important to capture watersheds with inputs from more concentrated livestock operations and highly urbanized landscapes to build a more complete picture of watershed-scale AMR dynamics. Therefore, a primary objective for the East Fork Little Miami pilot watershed study, apart from understanding of watershed scale AMR dynamics in this system, is to establish measurement protocols, sampling design parameters, and reporting guidelines that will facilitate data aggregation across studies as more watersheds are assessed.

### 3.2 AMR indicators for SWAM effort

The types of analyses that will be employed for the SWAM effort include a combination of culture-based and molecular-based techniques with indicator selection based on importance and relevance for human, animal, and environmental health. For culture analysis, *E. coli*, *Enterococcus* spp., and *Salmonella* spp. were selected as priority organisms for AMR monitoring in water based on what NARMS already assesses for food, animals, and humans as well as their environmental relevance (Nyirabahizi et al., 2020; Zhao et al., 2020; Yin et al., 2021). *E. coli* and *Enterococcus* are recommended fecal indicators for surface waters (U.S. Environmental Protection Agency (EPA), 2009, 2014), as well as sentinel organisms used by NARMS to monitor carriage and emergence of ARGs that could be transferred to both gram-negative and gram-positive pathogens (Ge et al., 2020). *Salmonella* is an important zoonotic pathogen (Alakomi and Saarela, 2009) that is systematically monitored by NARMS in human clinical isolates, outbreaks, retail meats, and food-producing animals.

Quantitative concentrations of ARB (counts or most probably number (MPN)) were deemed necessary since they add significant value to the analysis of AMR in surface waters for those indicators that are anticipated to be at sufficient density for quantification. Knowing the number of cultivable ARB can be used to: (1) compare magnitudes across sites/studies, (2) determine elevated risk with respect to background levels, (3) quantify risk using QMRA models; and (4) characterize gradients across land use. Therefore, *E. coli* and *Enterococcus* analysis will include colony counts and quantification of both total isolates and isolates resistant to select antibiotics (cefotaxime for *E. coli* and vancomycin for *Enterococcus*). A subset of resistant isolates will undergo species confirmation and subsequent whole genome sequencing (WGS). Given the variable and typically low numbers of *Salmonella* found in surface waters, a selective enrichment method will be utilized to determine presence or absence of *Salmonella* and to obtain isolates in pure culture in the presence of other bacteria. All *Salmonella* isolates will undergo WGS and, if possible, NARMS standard susceptibility testing. Quantification of antimicrobial susceptible *E. coli* and *Enterococcus* and obtaining isolates of *Salmonella*, *E. coli* and *Enterococcus* from surface waters will allow the SWAM effort to fit within the existing NARMS reporting framework as an environmental component moving toward a One Health assessment of AMR.

While culturing select priority organisms fits within the typical NARMS framework, given the complexity and diversity of the environmental microbiome, the inclusion of targeted molecular techniques can be used to provide a more expansive characterization of AMR in surface waters. The molecular methods to analyze environmental AMR will consist of quantification of ARGs, *intI1*, fecal source indicators, and other related genes and bacterial isolates using quantitative polymerase chain reaction (qPCR)/droplet digital PCR (ddPCR), metagenomics, and WGS. qPCR/ddPCR data will provide a quantitative assessment of ARGs, *intI1*, fecal indicators, and other genes of interest that are present across a microbial population which can inform models that impart information about AMR trends, hot spots, and/or reservoirs within surface waters. Furthermore, for those research efforts that cannot conduct extensive culture-based approaches, qPCR/ddPCR methods allow for the exploration of relationships between molecular fecal indicators and ARGs within a particular environment/microbial population.

Similarly, metagenomics will help identify types and sources of AMR contamination (animal production, agriculture, health care/human, etc.) by characterizing the resistome of the entire microbial community in surface waters (Mendes et al., 2017; de Abreu et al., 2020; Franklin et al., 2021). Metagenomics is also valuable for possibly selecting additional culture and/or molecular indicators, providing a more robust characterization of baseline contamination levels and differentiating risky ARGs from the background endogenous resistome. WGS together with *in silico* characterization of ARGs, plasmids, sequence types, and virulence factors can be employed to describe bacterial characteristics with much greater breadth and precision than phenotypic analysis alone (McDermott and Davis, 2021). WGS is also critical for detecting relatedness among isolates from different sampling locations including potential source or exposure areas, and it can be used to associate resistance with virulence and mobility traits to support risk assessment. Together, this array of methodologies will support a robust assessment of AMR dynamics at both the watershed and national scales for risk assessment as well as integration into existing NARMS monitoring programs.

Analysis of antibiotics was also considered as an important element to the evaluation of AMR and possible drivers of AMR in surface waters. The selection of antibiotics to analyze within surface waters should be based on antibiotic usage in humans and animals with a focus on high priority antibiotics like fluoroquinolones, sulfonamides, tetracyclines, trimethoprim, and aminoglycosides. While beta-lactams and the bacteria resistant to them (e.g., ESBL *E. coli*) are of highest priority and deemed critically important in human medicine, these antibiotic compounds are highly unstable in the environment and rarely found intact, especially in surface waters, due to the beta-lactam ring that can be opened by beta-lactamases (enzymes carried by certain bacteria) and/or by chemical hydrolysis (Christian et al., 2003; Huijbers et al., 2019). Even though the importance of analyzing for antibiotics was highlighted and discussed during the development of the SWAM effort, it was not included in the final designs of the watershed and national scale studies due to various reasons (e.g., cost and manpower constraints, concerns of what antibiotics to select, etc.), but may be revisited later.

### 3.3 Analytical method selection for SWAM AMR indicators

For method development and evaluation, utilization of standard methods when possible was deemed a high priority to ensure comparability of this effort with similar environmental monitoring efforts (World Health Organization, 2021; Liguori et al., 2022). Since various sampling and laboratory methods are used by different researchers, the SWAM environmental working group aimed to determine optimal method(s) for AMR analysis in surface waters that will provide comparative data across studies. As a result, standard methods were compared with those methods commonly used for analysis of AMR in surface waters, with final selection of methods based on their adaptability within the requirements and limitations of the SWAM effort as well as how well they aligned with similar water monitoring projects to create consistency across efforts. Any modifications to these methods occurred because they were deemed beneficial and/or necessary to support study objectives.

**FIGURE 2:**
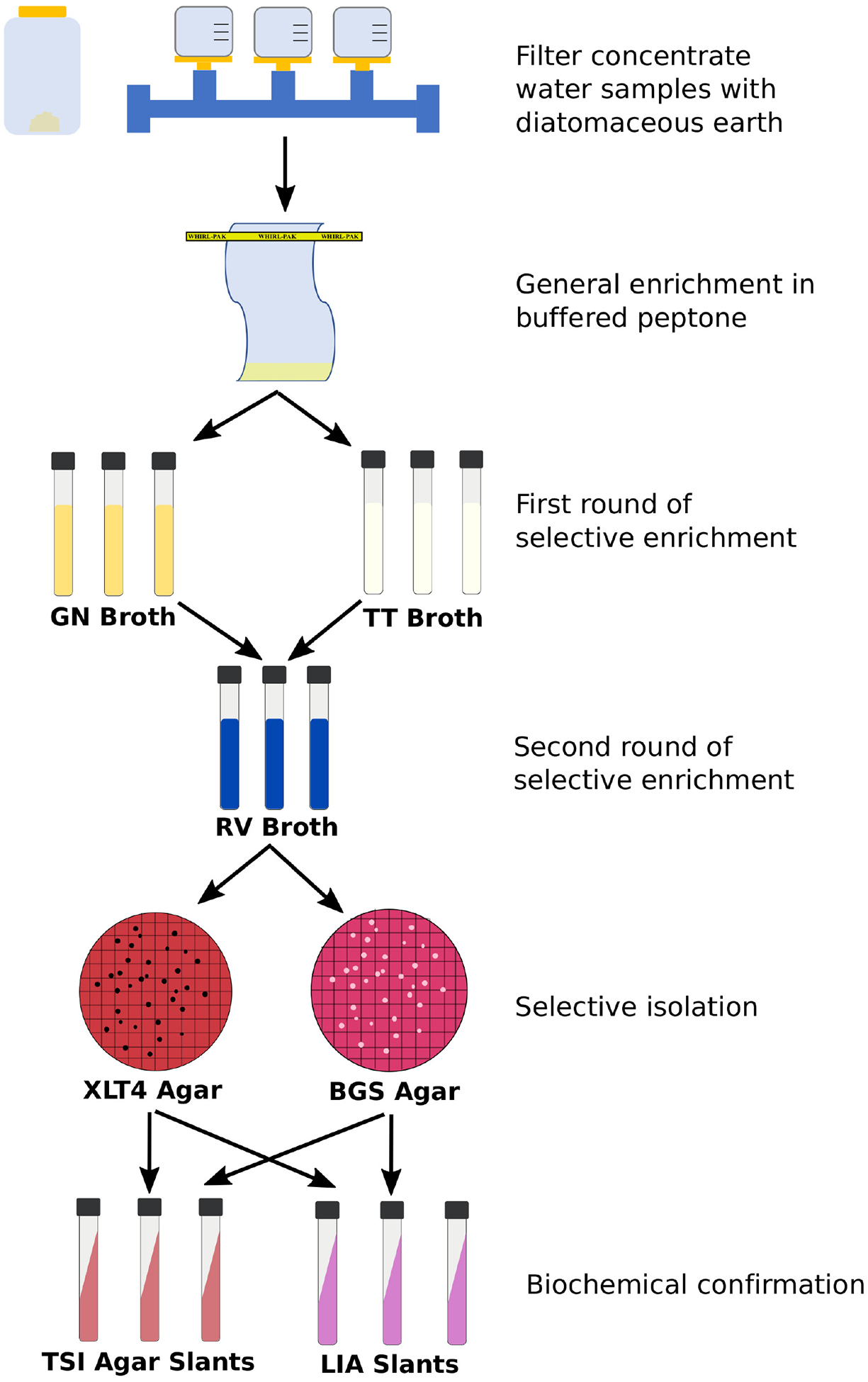
General flow chart of *Salmonella* enrichment and isolation procedure. Protocols for the filtration via the Modified Standard Method 9260.B2 and selective enrichment can be found at: dx.doi.org/10.17504/protocols.io.rm7vzy72xlx1/v2 and dx.doi.org/10.17504/protocols.io.kxygxz5q4v8j/v1, respectively. GN, Gram negative; TT, Tetrathionate; RV, Rappaport Vassiliadis; XLT4, Xylose lysine tergitol 4; BGS, Brilliant Green Sulfa; TSI, Triple Sugar Iron; LIA, Lysine Iron Agar.

Development of culture methods included evaluation of methods for the quantification of total and resistant *E. coli* and *Enterococcus* spp. and isolation of *Salmonella* spp. The culture methods that were considered for *E. coli* and *Enterococcus* spp. consisted of those commonly used and recommended for the quantification and isolation of these bacteria in surface waters, including standard methods recommended by WHO, EPA, and ASTM International (Table 4) with EPA 1603 and EPA 1600 selected for *E. coli* and *Enterococcus* spp., respectively. These methods were modified to perform susceptibility testing with cefotaxime for *E. coli* and vancomycin for *Enterococcus* spp. Method evaluation for *Salmonella* included considerations of different water volumes and comparisons of filtration and/or concentration techniques to optimize the recovery of low and sporadic levels of these bacteria in surface waters (Sharma et al., 2020; Kraft et al., 2023). Additionally, different selective enrichments, agars, and identification methods for *Salmonella* isolates (culture recovery versus rapid screening) were compared. The *Salmonella* method selected for this effort was based off the modified Standard Method 9260.B2, which has been used extensively to analyze surface waters in the southeastern U.S. (Meinersmann et al., 2008; Cho et al., 2022; Kraft et al., 2022). This method involves filtration utilizing perlite (in place of diatomaceous earth) to capture the bacterial cells, a general enrichment to revive injured cells, selective enrichments, and plating on selective media (Figure 2).

Method development for molecular techniques included comparisons of different water volumes, filtration techniques, DNA extraction kits, whole cell standards, and DNA standards. Having sufficient volumes of water and a DNA extraction kit that provided adequate amounts of high-quality DNA was deemed a high priority for the success of subsequent molecular work. Due to the lack of standardization for molecular techniques, including qPCR, ddPCR, WGS, and metagenomics, this work focused on having quality control measures at each step of sample processing to account for any processing variability. QA/QC guidelines will follow the Minimum Information for Publication of Quantitative Real-Time PCR Experiments (MIQE) guidelines (Bustin et al., 2009).

Although recommendations and guidelines for WGS and shotgun metagenomic data are currently limited for environmental studies, factors that are important across all next generation sequencing (NGS) approaches include data quality metrics such as average Q scores, sequence complexity distributions, contamination, number of ambiguous bases, sequence length, coverage and N50s for assembly. A minimum coverage, ranging from 30X for *Salmonella* to 40X for *E. coli*, will be targeted for all WGS experiments (Timme et al., 2020). For metagenomic studies, ‘coverage’ is a far more complicated subject because hundreds or thousands of distinct genomes may be present in any particular sample. Recommendations for depth of sequencing will vary by matrix and the overall aim of the study (Rodriguez-R and Konstantinidis, 2014). For taxonomic composition and AMR gene profiling, work has shown that the required depth of sequencing varies significantly by matrix (Gweon et al., 2019). The complexity and diversity of microbiomes in a sample, the interest to characterize the less abundant organisms, and sequencing cost were considered in deciding the depth of sequencing for the shotgun metagenomic sequencing. A shotgun metagenomics approach will be used to characterize the microbiome and to index the full complement of environmental AMR genes in the surface water samples. In addition to shotgun metagenomic sequencing, sequencing of culture enrichments from surface waters, known as quasimetagenomics, will be performed to characterize ARGs present in less abundant organisms. Preliminary studies by this NARMS surface water sampling initiative (Ottesen et al., 2022; Kocurek et al., 2024) have demonstrated that quasimetagenomic data could identify as many as 30% of critically important AMR genes (Table 5) from surface water samples while metagenomic data without enrichment only detected 1% of these AMR genes in the same samples at the same sequencing depth.

All sequencing reads will go through quality control steps to remove adaptors, low quality and complexity sequences using Trimmomatic (Bolger et al., 2014) prior to analysis. A combination of read-based/assembly-free and assembly-based approaches will be used for taxonomic and resistome profiling. For screening environmental metagenomes for ARGs, standalone databases containing functionally verified genes, such as CARD, NCBI’s AMRFinderPlus, and ResFinder (Feldgarden et al., 2019; Bortolaia et al., 2020; Feldgarden et al., 2021; Alcock et al., 2023) and predictive models, like DeepARG (Arango-Argoty et al., 2018) were examined for maximum coordination of gene nomenclature. Overall, the success of the molecular analysis is dependent on important considerations, like consistent quality control measures, metadata, data storage and sharing, as well as coordination of results from PCR, metagenomic, and WGS data.

### 3.4 Data management for SWAM effort

Given the large scope of the SWAM effort, the planning and management of metadata needed to be carefully considered. To identify and guide metadata needs, a conceptual schematic of the project scope was developed (Figure 1). The project was divided into the following categories based on setting and activity: Sample site, *in situ* measurements, weather and climate, sample collection, sample transportation, primary sample processing, culturing, metagenomics, targeted gene assays, water chemistry, and isolate WGS. This categorized approach was helpful as it segregated the development of the metadata standard into manageable sections.

Since this environmental study will include metagenomic and microbiome sequence data, the MIxS metadata standard, which is implemented by NCBI, will be used to facilitate ease of data submission. The MIxS, or Minimum Information about any (x) Sequence, standard is a metadata framework established and maintained by the Genomic Standards Consortium (Yilmaz et al., 2011). MIxS provides a standardized format for annotation of sample attributes through a series of environmental packages, including core terms as well as setting-specific checklists. One of the main points of emphasis within the MIxS framework is the re-use of existing terms from other environmental packages, when appropriate, to promote interoperability as well as to minimize metadata term maintenance efforts. Therefore, current MIxS environmental packages were examined to identify terms that could be reused for the metadata standard associated with this study, and currently includes 24 reused MIxS terms. A draft metadata sheet is presented in the Appendix 1.

To maximize the impact of the contextual information contained in this research study, ontological terms and definitions were utilized whenever possible. The current metadata standard draft includes 12 ontological terms. These terms include geographic location descriptors from Gazetteer ontology (GAZ), general biological and microbiological terms from the National Cancer Institute Thesaurus (NCIT), and phenotypic and microbiological terms from the Ontology of Prokaryotic Phenotypic and Metabolic Characters (MICRO) to name a few. The Ontology Lookup Service, maintained by EMBL-EBI and the Open Biological and Biomedical Ontology (OBO) Foundry were invaluable in finding existing ontological terms to define and describe certain attributes in the metadata standard.

## 4 Discussion

### 4.1 Next steps

With the preliminary planning, decision-making, method development, and method evaluation for the SWAM effort complete, the next steps for this effort are completion of the yearlong watershed scale study and the national scale study that will span a two-year time frame. During the East Fork Watershed study, thirty-five sites throughout the watershed will be sampled every three weeks with a few locations upstream and downstream of point sources being sampled weekly. This study will not only provide an opportunity to test the culture and molecular methods with a variety of sampling locations during base flow and various weather conditions (rain events, snow, snow melt, etc.) but will also provide information about the temporal variation of AMR, assist in assessing possible drivers of AMR, inform exposure risk assessment, and/or identify critical control points at a watershed scale.

The national-scale study for the SWAM effort will utilize the U.S. EPA NRSA survey that will be executed in 2023–2024. This national scale assessment of rivers and streams occurs every five years over a two-year time frame (sampling during May – September) and includes approximately 2,000 sites (about 1,000 sites per year). Sites are sampled only during base flow conditions, and most sites are only visited once, except for 10% that are revisited as a quality control measure. Since the NRSA survey collects a wide variety of water quality indicators to assess the ecological condition of surface waters nation-wide, this national-scale study will build off the trends previously identified by Keely et al. (2022) and provide additional information about the spatial variation of AMR across the nation as well as how water quality parameters may correlate with AMR indicators.

### 4.2 Future directions for SWAM

Once the watershed scale and national scale studies are completed, the SWAM effort will have generated a library of isolates (*Salmonella*, *Enterococcus*, and *E. coli*) that will be compared and cross-referenced with the NARMS isolate libraries to explore interconnections between human, animal, and environmental compartments at local, regional, and national scales. Assessments of what was successful and/or feasible during the watershed- and national-scale studies will guide the development of the national environmental monitoring program as well as recommendations for how to perform additional watershed-scale studies. Other needs or questions that remain to be addressed can be added during subsequent watershed-based and national-scale studies. Having validated standard frameworks for environmental monitoring of AMR will facilitate data aggregation across these studies as additional watershed- and national-scale studies are performed.

The SWAM effort will be a significant step forward for environmental monitoring and the assessment of AMR from a One Health perspective, allowing direct comparison of surface water isolates and metagenomes with existing NARMS isolate libraries. This effort will produce standard measurement protocols, sampling design parameters, and reporting guidelines for monitoring AMR in surface waters at both the watershed and national scale. The protocols from this effort could also be utilized by other researchers in their own surface water studies (e.g., additional watershed scale studies), which can then be integrated into larger assessments/meta-analyses to address deeper questions about AMR dynamics. Overall, the unique data set on surface waters produced by this effort will provide a One Health assessment of AMR to support the NARMS monitoring program and create a framework for environmental monitoring programs at national and international scales.

## Supplementary Material

Supplemental Material 1

## Figures and Tables

**TABLE 1:** List of subgroups and their respective tasks for developing an environmental monitoring effort for antimicrobial resistance.

Subgroup name	Tasks
End use of data	Provided an interface with NARMS program to ensure integration with existing NARMS reporting structures and that data collected meets user needs.
Sampling design	Developed a statistically valid sampling design that includes: Yearlong watershed study in East Fork Watershed (southeast Ohio) to assess AMR dynamics within a watershed. National study utilizing EPA’s National Rivers and Streams Assessment to assess AMR trends at a national scale.
Metadata	Identified metadata needs and developed a system for managing data. Metadata needs were based around the following categories: sample site, *in situ* measurements, weather and climate, sample collection, sample transportation, primary sample processing, culturing, metagenomics, targeted gene assays, water chemistry, and isolate WGS. See Supplementary material for a draft metadata sheet for this effort.
Field sampling	Developed field sampling protocols for collection of surface water samples during watershed and national studies. Protocols included aseptic techniques appropriate for samples intended for culture and molecular work.
Culture	Identified culture targets and identified/developed culture methods for the analysis of the selected targets. Selected culture targets and methods included: *E. coli* and ESBL *E. coli* via modified mTEC Method (modification of EPA 1603) *Enterococcus* spp. and vancomycin resistant *Enterococcus* spp. via modified mEI Method (modification of EPA 1600) *Salmonella* via Standard Method 9263B
Molecular	Identified molecular targets and identified/developed molecular methods for the analysis of the selected targets. Molecular work includes: Metagenomics Whole Genome Sequencing of all *Salmonella* isolates and resistant *E. coli* and *Enterococcus* isolates Targeted gene analysis of select antimicrobial resistance genes, including, but not limited to, (*bla*_CTX-M_, *vanA****,*** *sulI****,*** and *tetA*)*,* integrases (e.g., *intI1*), fecal indicators, and mobile genetic elements.

**TABLE 2:** Phases of the SWAM effort to implement the national monitoring system.

Phase	Objective
1 Method development and in lab validation	Development of study and sampling design. Comparison of methods for each target. Selection of methods.
2 Single watershed pilot study	Perform a yearlong watershed study to validate the methods selected in Phase 1 and serve as a demonstration study for future watershed scale studies.
3 Probabilistic national study	Perform the pilot national study utilizing the methods selected during Phase 1 and validated during Phase 2.
4 Finalized national monitoring program	The study and sampling design for a national monitoring program finalized and validated by Phase 3.
5 Additional focused studies	Additional studies, including watershed scale, to utilize the methods developed by this effort to answer additional research and monitoring questions.

**TABLE 3:** NARMS surface water pilot goals compared to different study designs.

NARMS surface water pilot goals	National probabilistic survey (NRSA)	Demonstration watershed study (EFLMR)	Multiple watershed studies
Determine spatial extent of AMR in surface waters	***	*	**
Determine temporal variation in AMR	*	***	***
Evaluate environmental correlates of AMR	***	**	***
Assess One Health environmental connections	*	**	***
Develop environmental risk assessment models	*	***	***
Assess environmental sources/drivers/attenuators of AMR	**	***	***
Assess control/intervention approaches	*	***	***

Note the complementarity of the national probabilistic survey and the watershed study.

*** *Highly effective*;

** *Somewhat effective*;

* *Somewhat useful*.

**TABLE 4:** Methods considered for the enumeration of *E. coli* and *Enterococcus* from water.

*E. coli*		
Media	Mechanism	Method/Reference
TBX	Membrane filtration	WHO Tricycle (World Health Organization, 2021)
mTEC	Membrane filtration	EPA 1603 (U.S. Environmental Protection Agency (EPA), 2014)
mI	Membrane filtration	EPA 1604 (U.S. Environmental Protection Agency (EPA), 2002)
Colilert	MPN	Standard Method 9223B (Standard Methods Committee of the American Public Health Association, American Water Works Association, and Water Environment Federation, 2012)
*Enterococcus*		
Media	Mechanism	Method/Reference
mEI membrane-Enterococcus Indoxyl-β-d-glucoside agar	Membrane filtration	EPA 1600 (U.S. Environmental Protection Agency (EPA), 2009)
Enterococcosel agar	Membrane filtration	Royal Society of Chemistry (Corry et al., 1996)
CHROMagar Orientation	Membrane filtration	Merlino et al. (1996)
Enterolert	MPN	ASTM D6503 (ASTM, 2000)

**TABLE 5:** List of genes encoding resistance to critically important antimicrobial agents identified by the National Antimicrobial Resistance Monitoring System (NARMS).

Macrolides	β-lactams	Colistins	Quinolones/Fluoroquinolones
23S rRNA A2075G point mutation	*bla*_CMY-2, 4, 30, 43_’	*mcr-1.1, 1.2, 3.1, 3.24*	*aac(6’)-Ib-cr*
*acrB* R717L point mutation	*bla*_CTX-M1, 2, 3, 8, 9, 14, 15, 27, 32, 55, 65, 101, 104, 165, 190_		*qnrA1, B1, B2, B4, B6, B7, B9, B19, B38, S1, S2, S4, S13, VC1*
*ere(A)*	*bla*_FOX-5_		*gyrA* *
*erm42, A, B, T*	*bla*_KPC-2, 4_		*gyrB* S464F point mutation
*mefB, C*	*bla*_NDM-1_		*msr(C)*
*mphA, B, E, G*	*bla*_SHV-7,12, 30_		*oqxA, B*
*msr(E)*	*bla*_TEM-15, 207_		*parC* **
			*parE* ***
			*qepA, A1*

Genes from *Salmonella*, *Escherichia coli*, *Campylobacter*, and *Enterococcus* are reported together.

* *gyrA* Point Mutations: D87G, D87N, D87Y, D90Y, P104S, S83A, S83F, S83L, S83Y, T86A, T86I, T86K, T86V.

** *parC* Point Mutations: A56T, E84G, S801I, S80R.

*** *parE* Point Mutations: D475E, I529L, L416A.

## Data Availability

The raw data supporting the conclusions of this article will be made available by the authors, without undue reservation.
